# Transcatheter Arterial Embolization in Acute Non-Variceal Gastrointestinal Bleedings: A Ten-Year Single-Center Experience in 91 Patients and Review of the Literature

**DOI:** 10.3390/jcm10214979

**Published:** 2021-10-27

**Authors:** Federico Fontana, Filippo Piacentino, Christian Ossola, Andrea Coppola, Marco Curti, Edoardo Macchi, Giuseppe De Marchi, Chiara Floridi, Anna Maria Ierardi, Gianpaolo Carrafiello, Sergio Segato, Giulio Carcano, Massimo Venturini

**Affiliations:** 1 Diagnostic and Interventional Radiology Department, Ospedale di Circolo, ASST dei Sette Laghi, 21100 Varese, Italy; federico.fontana@uninsubria.it (F.F.); filippo.piacentino@asst-settelaghi.it (F.P.); andrea.coppola@asst-settelaghi.it (A.C.); edoardo.macchi@asst-settelaghi.it (E.M.); giuseppe.demarchi@asst-settelaghi.it (G.D.M.); massimo.venturini@uninsubria.it (M.V.); 2 School of Medicine and Surgery, Università degli Studi dell’Insubria, 21100 Varese, Italy; curti.marco.33@gmail.com (M.C.); giulio.carcano@uninsubria.it (G.C.); 3 Department of Radiology, University Hospital “Umberto I—Lancisi—Salesi”, 60100 Ancona, Italy; chiara.floridi@gmail.com; 4 Department of Radiology, Foundation IRCCS Ca’ Granda-Ospedale Maggiore Policlinico, University of Milan, 20122 Milan, Italy; annamaria.ierardi@policlinico.mi.it (A.M.I.); gianpaolo.carrafiello@unimi.it (G.C.); 5 Gastroenterology Department, Ospedale di Circolo, ASST dei Sette Laghi, 21100 Varese, Italy; sergio.segato@asst-settelaghi.it; 6 Surgery Department, Ospedale di Circolo, ASST dei Sette Laghi, 21100 Varese, Italy

**Keywords:** gastrointestinal bleeding, trans-arterial embolization, embolic agents

## Abstract

Objective: To report the safety and efficacy of trans-arterial embolization (TAE) for upper gastrointestinal bleeding (UGIB) and lower gastrointestinal bleeding (LGIB) due to different etiologies in 91 patients for ten years. Methods: A retrospective analysis of GIB treated between January 2010 and December 2020 was performed. TAE was performed using different embolic agents (coils, particles, glue, gelatin sponge, and EVOH-based agents). Technical success, secondary technical success, clinical success, and complications were evaluated. Results: Technical success was achieved in 74/91 (81.32%) patients. Seventeen patients (18.68%) required re-intervention. Secondary technical success was achieved in all cases (100.0%). Clinical success was achieved in 81/91 patients (89.01%). No major complications were recorded; overall, minor complications occurred in 20/91 patients. Conclusions: TAE is a technically feasible and safe therapeutic option for patients with GIB from a known or blind anatomic source where endoscopic therapy has failed or is deemed unfeasible.

## 1. Introduction

Gastrointestinal bleeding (GIB) is one of the major causes of hospitalization, responsible for 1–2% of all admissions and burdened with a high death rate of up to 25% [1,2].

The incidence increases with age, occurring in 70% of patients older than 65 years [1], and with comorbidities, especially in those cases requiring blood thinners. Moreover, acute GIB complicates the clinical course of critically ill patients admitted with other primary diagnoses [3,4].

The main causes of GIB include peptic ulcer disease, esophagitis, gastritis and duodenitis, varices, angiodysplasia, diverticular disease, malignancy, arteritis, aortoenteric fistulas, inflammatory bowel disease, and colitis [4,5,6].

The diagnostic-therapeutic care pathway for GIB varies based on the gastrointestinal tract involved, with important differences in terms of epidemiology, clinical management, and prognosis [1]. Many different treatment strategies can be used—e.g., conservative endoscopic therapy, transarterial embolization (TAE), and surgery; therefore, a multidisciplinary approach to the problem is essential [7].

In the majority of cases (70–80%), GIB will resolve spontaneously with supportive measures alone; in the remaining cases, further intervention is required, involving gastroenterologists, surgeons, and interventional radiologists [8].

Esophagogastroduodenoscopy is considered the gold standard for patients with massive upper gastrointestinal bleeding (UGIB), with reported sensitivity and specificity up to 98% and 100%, respectively [9,10]. For lower gastrointestinal bleeding (LGIB), colonoscopy is the preferred choice for most patients given its high diagnostic performance, varied therapeutic capabilities, and low complication rate [11]. However, the endoscopic approach is not always feasible because of a variety of challenges, such as variable availability of the endoscopist or the poor amount of time to obtain an adequate bowel preparation in the most serious cases [12].

Over the years, interventional radiology hemostasis techniques have surged as an important treatment in GIB, combining excellent technical and clinical success rates with a low risk of complications [13,14,15,16].

Surgical intervention should be considered only for patients with uncontrolled severe bleeding or multiple failed nonsurgical treatment attempts [17].

Several studies have recently shown the safety and efficacy of TAE with the use of advanced tools and techniques for UGIB and LGIB, but to our knowledge, most of them have included a limited number of patients [2,4,18,19,20,21,22,23,24,25,26,27]. Coil embolization is the most used technique worldwide to treat GIB, avoiding bowel ischemic complications [2,4].

The aim of this ten-year retrospective study was to report the safety, efficacy, and clinical outcome of super selective TAE for UGIB and LGIB due to different etiologies comparing our data with those of the current literature.

## 2. Materials and Methods

### 2.1. Patient Selection and Study Design

In this single-center retrospective study, from January 2010 through December 2020, consecutive patients from internal medicine and general surgery departments and intensive care units who underwent TAE to control arterial GIB in the interventional radiology unit of a tertiary university center were identified; patients with venous variceal hemorrhages were excluded.

Written informed consent including consent to publish anonymized data was obtained from all patients. Institutional review board approval was not required because of the retrospective nature of the work.

### 2.2. Endpoints

The primary endpoints were to report technical and clinical success of TAE in GIB; the secondary endpoints were major and minor complications. Our collected data were compared with the literature.

### 2.3. Angiography and Embolization Procedure

Indication for TAE was given in a multidisciplinary team setting including at least radiologist, surgeons, endoscopist, and an emergency care or intensive care clinician, according to patient origin (emergency care department or inpatients, respectively). Either acute massive/recurrent GIBs where endoscopy had failed or was not feasible endoscopically were included. In most patients with clinical suspicion for sub/acute bleeding, CT angiography (CTA) including at least a basal, an arterial, and a venous phase was obtained prior to digital subtraction angiography (DSA) in order to localize the bleeding site and to evaluate the vascular anatomy.

TAE was always attempted in the case of CTA and/or angiographic evidence of active contrast extravasation, pseudoaneurysm, or abnormal vascular blush. Following TAE, repeat angiograms were performed to determine the adequacy of TAE and to assess collateral vessels.

All procedures were performed in a single angiography suite under fluoroscopic guidance (AlluraXper FD20, Philips Healthcare, Best, The Netherlands). The contrast medium used was iodixanol (Visipaque, GE Healthcare, Cork, Ireland).

All TAE were performed using a transfemoral approach after local anesthesia with 10 mL injection of 2% Mepivacaine (Angelini, Roma, Italy) under peri-interventional observation with continuous monitoring of blood pressure, electrocardiographic parameters, arterial oxygen saturation, and, depending on the patient’s hemodynamic status, the presence of an anesthesiological team.

Diagnostic angiograms were usually carried out using 5-Fr Cobra or Simmons catheters (Cordis, Miami, FL, USA), probing the celiac trunk and the superior mesenteric artery (SMA) for UGIB and the SMA and the inferior mesenteric artery (IMA) for LGIB. A superselective arteriography and TAE were performed using a 0.027-inch microcatheter (Progreat, Terumo; Tokyo, Japan).

The embolic agents used, according to operator preference, embolization site, vessels, patient characteristics, and store availability, were microcoils (Concerto^®^, Medtronic B.V, Heerlen, The Netherlands); 3 hydroxyl methyl acrylic acid gelatin microspheres (BeadBlock^®^, Biocompatibles, UK); gelatin sponges (Gelfoam, Pharmacia & Upjohn; Kalamazoo, MI, USA); liquid agents including n-butyl 2-cyanoacrylate (NBCA) glue (Glubran^®^, GEM, Viareggio, Italy); and ethylene-vinyl alcohol copolymer (EVOH) (Onyx^®^, MicroTherapeutics, Inc., Irvine, CA, USA; Squid^®^, Emboflu, Gland, Switzerland).

### 2.4. Outcomes Measures

The following data were collected: mean age, age range, gender distribution, involved arteries, percentages of emergency procedures, embolizing agents used, contrast medium amount, technical success, secondary technical success, clinical success major/minor complications, and mean follow-up.

Technical success, peri-procedural complications, clinical success (imaging/clinical data at 1 month), and post-procedural complications were assessed according to international standard guidelines [28]. Technical success was defined as an immediate angiographic result of complete occlusion of target vessel(s) after a single procedure; secondary technical success was defined as complete occlusion of target vessel(s) after re-intervention; clinical success was defined by the combination of technical success and the lack of bleeding within 30 days after the procedure.

According to standards of practice guidelines of the Society of Interventional Radiology [19], major complications were defined and classified as follows. Grade 1: complications that require therapy and minor hospitalization (<48 h); grade 2: complications that require major therapy, unplanned increase in level of care, and prolonged hospitalization (>48 h); grade 3: complications that require permanent adverse sequelae; grade 4: death. Minor complications were defined as complications without sequelae or requiring nominal therapy or short hospital stay for observation (generally overnight).

Embolized vessels, causes of bleeding, and embolizing agents utilized were also evaluated.

## 3. Results

Ninety-one consecutive patients with GIB were submitted to TAE. There were 52 (57.1%) men and 39 (42.9%) women, with a mean age 71.3 (range: 23-90) years old.

TAE was performed in 43/91 (47%) UGIB and in 48/91 (53%) LGIB.

In 21/91 patients, TAE was performed after unsuccessful or failed endoscopic interventions; of these, 12/21 were patients for UGIB, and 9/21 were patients for LGIB.

The amount of contrast medium in the angiographic procedure varied from 80 to 100 mL.

**Figure 1:**
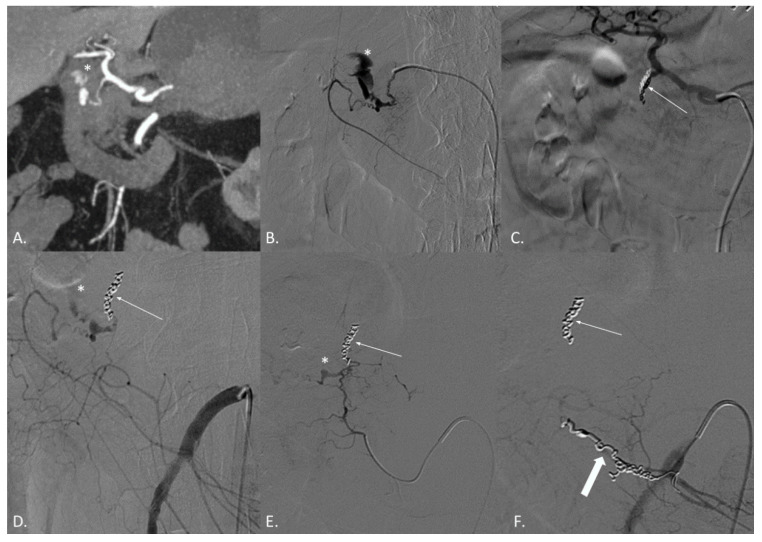
(**A**–**F**) CT angiography (CTA) and digital subtraction angiography (DSA) of a 74-year-old male patient suffering from active bleeding of a duodenal peptic ulcer successfully treated with coil embolization both from the celiac trunk and the superior mesenteric artery (SMA)—“sandwich technique”. (**A**) CTA demonstrates active endoluminal bleeding (*) from the gastroduodenal artery (GDA). (**B**) DSA obtained with catheterization of the GDA confirms CTA findings. (**C**) DSA obtained with catheterization of the celiac trunk after coils and gelfoam embolization of the GDA (thin arrow). (**D**) DSA obtained with catheterization of the SMA demonstrating residual active bleeding (*) from the inferior pancreaticoduodenal arteries (iPDA). (**E**) DSA obtained with catheterization of the iPDA confirms the active bleeding (*). (**F**) DSA obtained with catheterization of the SMA after coil embolization of the iPDA (thick arrow). No active bleeding evidence.

The most commonly embolized vessel was the gastroduodenal artery (GDA; *n* =36, 39.6%), followed by the superior mesenteric artery (SMA; *n* = 30, 32.97%), the inferior mesenteric artery (IMA; *n* = 18, 19.78%), and the left gastric artery (LGA; *n* = 7, 7.69%) Figure 1.

**Figure 2:**
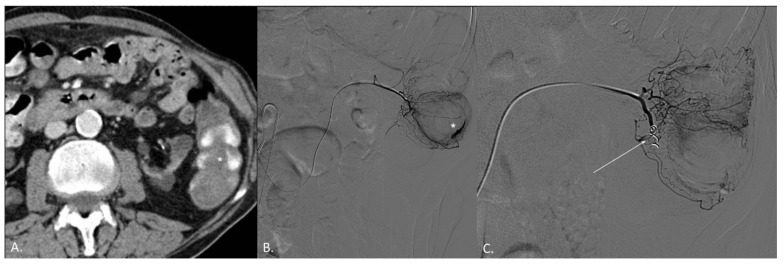
(**A**–**C**) CT angiography (CTA) and digital subtraction angiography (DSA) of an 82-year-old female patient. (**A**) CTA demonstrates active endoluminal bleeding (*) from left colon angiodysplasia. (**B**) DSA obtained with catheterization of the left colic artery (LCA) confirms CTA findings. (**C**) DSA obtained with catheterization of the LCA after coil embolization. No active bleeding evidence.

**Figure 3:**
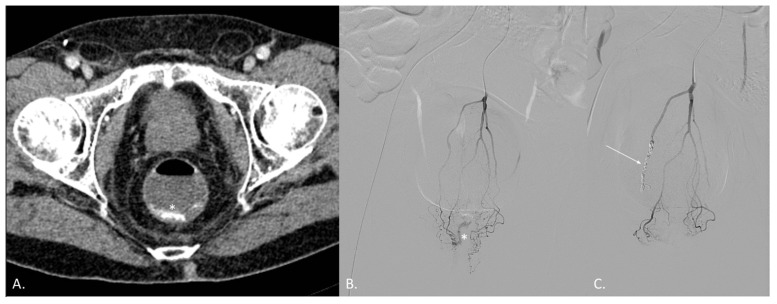
(**A–C**) CT angiography (CTA) and digital subtraction angiography (DSA) of a 68-year-old male patient known for radiation-induced proctitis after prostate cancer treatment. (**A**) CTA demonstrates active endoluminal bleeding (*) from the superior hemorrhoidal artery (SHA). (**B**) DSA obtained with catheterization of the inferior mesenteric artery (IMA) confirms CTA findings. (**C**) DSA obtained with catheterization of the IMA after coil embolization of the SHA (arrow). No active bleeding evidence.

Angiodysplasia (*n* = 20, 21.98%) was the most common etiology of GIB Figure 2 followed by duodenal ulcer (*n* = 15, 16.48%), undetermined etiology (*n* = 13, 14.29%), diverticular disease (*n* = 13, 14.29%), malignancy (n = 10, 10.99%), gastric ulcer (*n* = 8, 8.80%), pancreatitis (*n* = 6, 6.59%), iatrogenic (*n* = 4, 4.40%), and post-traumatic (*n* = 2, 2.20%) Figure 3.

Coils alone were used to treat LGA in 2 patients, GDA in 25, SMA in 12, and IMA in 13 cases. In 30 cases, coils were used in association with other embolic agents to treat LGA in 3 cases, GDA in 8 cases, SMA in 15, and IMA in 4 cases. Glue was used in one patient with GDA bleeding while EVOH agents were used to treat LGA in two cases, GDA in two, SMA in one, and IMA in one case. Microparticles were used in two cases of SMA bleeding.

To summarize, coil embolization alone or associated with other embolic agents was performed in 90% of cases (82/91), while in 10% of cases (9/91) embolization was performed without coils. There was no significant difference in terms of complication rates between the two populations.

All procedures were performed in emergency; TAE was preceded by endoscopy in 21/91 (23.08%) cases (2 bleedings from LGA, 10 from GDA, 7 from SMA, and from 2 IMA).

All data concerning patients’ characteristics, etiology of bleeding, and embolic agents used are reported in Table 1.

### 3.1. Outcomes

Technical success was achieved in 74/91 (81.32%) patients. Seventeen patients (18.68%) required re-intervention. Secondary technical success was achieved in all cases (100.0%). Clinical success was achieved in 81/91 patients (89.01%): all 10 cases of clinical unsuccess were related to patient loss by multiorgan failure during post-procedural care, non-procedure-related. Mean follow-up was 22 ± 4 days.

### 3.2. Periprocedural Complications

No major periprocedural complication was recorded. The minor periprocedural complication rate was 21.98% (20/91 cases: fever in seven cases, puncture hematoma in five cases, temporary liver or renal function failure in five cases, and temporary abdominal distension/abdominal ache within 2 to 3 days after embolization in three cases).

## 4. Discussion

The present study, over a ten-year period, confirms TAE as a viable therapeutic choice for GIB with a low complication rate. CTA plays a pivotal role in the detection of the site and the cause and extent of bleeding, providing information about the vascular anatomy and any anatomical variants.

Gastrointestinal bleeding has socioeconomic significance; it is more frequent in the elderly population, often burdened by comorbidities, and is costly [29].

GIBs can be distinct in UGIB and LGIB in relation to the location of the bleeding site. Upper gastrointestinal bleeding refers proximal to Treitz; on the other hand, LGIB occurs distal to Treitz, including the small intestine, colon, and rectum [4]. Endoscopy is currently considered the diagnostic and therapeutic gold-standard procedure for UGIB with a sensitivity and specificity of 92–98% and 30–100%, respectively [8]. However, endoscopic hemostasis for patients with massive LGIB is problematic due to the procedure time and the need for accurate bowel preparation [18].

In this scenario, CT angiography (CTA) is a non-invasive high sensitivity and specificity method that allows rapid and accurate diagnosis of acute GIB and gives important information regarding the cause of bleeding and possible vascular anomalies and variants determining the surgical or interventional radiology (IR) approach [30].

Endovascular hemostasis has been widely adopted because of rapid positioning, safety, and efficiency [4]. Moreover, it can also reduce the probability of hemorrhaging patients having to undergo surgical operations [31].

This study includes a conspicuous number of patients, including both UGIB and LGIB (Table 2).

In our study, the technical success and clinical success rate were 81.3% and 89.0%, respectively, both comparable to those reported by previous studies, ranging from 92 to 100% and from 63 to 95.8%, respectively [2,4,26,27,32,33,34,35,36,37,38,39,40].

The rebleeding rate was 18.7%, similar to Shi et al. who reported a postoperative rebleeding rate within 30 days of 15.29% [4]. In our case, 7 out of 17 rebleeding cases (41.2%) were under anticoagulant therapy. Among clinical factors predicting rebleeding, coagulopathy has been advocated to adversely affect both the technical success rate and post-TAE mortality, with an increase in the odds ratio for clinical failure [40,41].

The diversity of blood supply origins, etiologies, and bleeding characteristics has led to differences in imaging and methods of embolization, so the distinction between upper and lower GI hemorrhage has great significance [4].

According to Loffroy et al. [40], and also in our experience, the main reason for UGIB (43/91 patients) was ulcer disease, which accounted for 34.88%, followed by malignancy and undetermined causes.

Based on existing clinical data [42], we performed radiological endovascular intervention in cases in which an endoscopic approach was not applicable or there was a technical failure of endoscopic hemostasis; in these cases, the presence of a metallic clip has resulted in a more efficient localization of the culprit vessel, increasing the efficacy of TAE and decreasing the risk of inadequate coil migration and unintentional hepatic embolization [43].

In cases of intermittent bleeding, “blind embolization” was required, often successfully; empiric or blind embolization is a matter of debate, even if in the main studies, no difference in terms of outputs has been reported [23,30]. Moreover, this approach can be used in both GDA and LGA because they are responsible for 80–90% of UGIB [14,43].

In patients with GDA bleeding, the “sandwich technique” was performed, with the embolization of both the GDA and the pancreatic-duodenal arches, to prevent bleeding arising from branches of the SMA [43,44,45,46]

In the present report, the main causes of LGIB (48/91 patients) were arteriovenous malformations such as angiodysplasia (35.41%), followed by diverticular disease and indeterminate causes.

There are several studies that have reported the technical and clinical outcomes of TAE for LGIB, and superselective embolization is one of the important factors because of the reduced vascular anastomoses compared to the upper gastrointestinal tract [16,47,48].

The choice of embolic material is an essential part of the interventional embolization process and is primarily influenced by the operator experience, the cause of bleeding, and the coagulation status [49].

In our experience, coils were employed alone in 52/91 (57.1%) patients and combined with other embolic agents in 30/91 (33.0%) patients. In previous reports, coils had a higher technical success rate both for UGIB and LGIB [49], and a reduced risk of rebleeding [13].

One of the complementary embolic agents employed in this study includes gelatin sponges; this is a temporary embolic agent mainly used for adjuvant embolization in the treatment of acute GIB [50].

One other embolic agent employed was microspheres, alone or in combination. Particulate embolic agents such as polyvinyl alcohol particles or microspheres are associated with a higher risk of bowel ischemia, so an accurate superselective embolization is needed [51].

Cyanoacrylates were used in this study alone or in combination in the management of UGIB in hemodynamically unstable patients or in cases with impaired coagulation [52,53].

Ethylene-vinyl alcohol copolymers are liquid, non-adhesive, radiopaque embolizing agents; are more manageable than glue due to their prolonged solidification time than cyanoacrylates [53,54,55]; and were employed in 6/91 patients.

Based on data from the literature, coils should be employed in association with other embolic agents to prevent rebleeding, and glue should be used more frequently in patients with impaired coagulation thanks to its better hemostasis [40,43]

This study does come with a number of caveats. First of all, our study was a single-center retrospective review with a small sample size, which precluded the determination of predictors of re-bleeding or mortality. In addition, this study did not assess late complications of TAE, because of the short-term follow up.

## 5. Conclusions

In our experience, in both UGIB and LGIB, coils alone or in combination with other embolizing agents have proved to be the most used embolizing agent in relation to their precision in deployment and therefore in controlling the risk of ischemic complications. In UGIB, other embolizing agents such as glue rather than EVOH-based agents can also be utilized in relation to the anatomical characteristics of this area.

In conclusion, TAE is a technically feasible and safe therapeutic option for patients with GIB where endoscopic therapy has failed or is deemed unfeasible.

## Figures and Tables

**Table 1:** Patients’ baseline characteristics.

Vessel Involved	LGA	GDA	SMA	IMA
cases	7	36	30	18
Sex				
Female	4	15	9	11
Men	3	21	21	7
Etiology				
Angiodysplasia	\	3	10	7
Diverticular disease	\	4	5	4
Undetermined	2	3	4	4
Post-Traumatic	\	2	\	\
Duodenal Ulcer	\	12	3	\
Gastric Ulcer	\	3	2	\
Pancreatitis	3	3	3	\
Iatrogenic	\	1	\	3
Malignancy	2	5	3	\
Preliminary CTA				
Positive	6	25	19	11
Negative	1	6	2	2
Not performed	\	5	9	5
Endoscopy	2	10	7	2
Embolic agents				
Coils	2	25	12	13
Coils + other embolic agent	3	8	15	4
Glue	\	1	\	\
EVOH agents	2	2	1	1
Microparticles	\	\	2	\
Re-bleeding	2	7	6	2

LGA: left gastric artery; GDA: gastroduodenal artery; SMA: superior mesenteric artery; IMA: inferior mesenteric artery. “\” stands for 0 cases.

**Table 2:** Literature review.

Author	Journal	Year	Number of Patients	UGIB	LGIB	Embolizing Agents	Technical Success (%)	Clinical Success
Muhammad et al. [2]	*Cureus*	2018	32	X	X	Coils + other embolic agents	96.9	71.9
Shi et al. [4]	*Medicine*	2017	88	X	X	Coils + other embolic agents	100	84.7
Park et al. [32]	*BJR*	2021	15	X	-	Coils + other embolic agents	93.3	93.3
Hermie et al. [33]	*Eur Radiol*	2020	85	-	X	Coils + other embolic agents	100	not reported
Bua-ngam et al. [34]	*Interv imaging*	2017	38	-	X	Coils + other embolic agents	92	63
Kaminskis et al. [35]	*World J Emerg Surg*	2017	25	X	-	Coils	not reported	not reported
Hongsakul et al. [36]	*Acta Radiol*	2014	70	X	-	Coils + other embolic agents	98.6	71.4
Anil et al. [37]	*Clin Radiol*	2012	15	X	-	Coils	100	86.6
Kohler et al. [38]	*World J Surg*	2014	54	X	X	Coils + other agents	100	81.8
Griffiths et al. [26]	*ANZ J Surg*	2016	103	X	-	Coils + other agents	92	not reported
Rossetti et al. [39]	*Int J Colorectal Dis*	2013	24	-	X	Coils + other agents	100	95.8
Hur et al. [27]	*JVIR*	2014	112	-	X	Coils + other agents	96.4	74.5
Loffroy et al. [40]	*Clin Gastroenterol Hepatol*	2009	60	X	-	Coils + other agents	95	71.9

“X “stands for gastrointestinal district treated.

## Data Availability

The data presented in this study are available in Table 1.
